# The Assessment of a Multifactorial Score for the Adaptability Evaluation of Six Poultry Genotypes to the Organic System

**DOI:** 10.3390/ani11102992

**Published:** 2021-10-18

**Authors:** Alice Cartoni Mancinelli, Simona Mattioli, Laura Menchetti, Alessandro Dal Bosco, Claudia Ciarelli, Monica Guarino Amato, Cesare Castellini

**Affiliations:** 1 Department of Agricultural, Environmental and Food Science, University of Perugia, Borgo XX Giugno 74, 06124 Perugia, Italy; acartonimancinelli@gmail.com (A.C.M.); alessandro.dalbosco@unipg.it (A.D.B.); claudi-aciarelli@libero.it (C.C.); cesare.castellini@unipg.it (C.C.); 2 Department of Veterinary Medicine, University of Perugia, Via San Costanzo 74, 06126 Perugia, Italy; laura.menchetti7@gmail.com; 3 Council for Agricultural Research and Economics—Animal Production and Aquaculture, Via Salaria 31, 00015 Roma, Italy; monica.guarinoamato@crea.gov.it

**Keywords:** chicken, organic system, daily weight gain, genotype, adaptability score

## Abstract

**Simple Summary:**

The choice of a suitable poultry genotype for the organic system is still an open question. Currently, poultry genotypes are mainly classified on the basis of their daily weight gain (DWG). However, it is important to underline that the organic system is characterized by the presence of outdoor space; therefore, the grazing capacity of chicken and other intrinsic characteristics are crucial aspects. Indeed, although DWG is an important parameter, it is probably not the only factor that should be considered to determine the adaptability of poultry to this system, in which the genotype could also have an important role. Accordingly, this study defines an adaptability score (AS) using a multifactorial approach in order to consider simultaneously different variables such as behaviors, plumage conditions, and body lesions. Moreover, the specific effect of DWG and genotype on the AS were evaluated. This allowed us to conclude that the DWG and genotype are connected, but the chicken genotype is the driving force that should be considered in order to evaluate poultry’s adaptability to organic systems.

**Abstract:**

This study aimed to develop an adaptability score (AS) for chicken strains, which includes behavioral, plumage conditions, and body lesion indicators through a multifactorial approach. A total of 600 male chickens from 6 poultry genotypes—Ranger Classic (R1), Ranger Gold (R2), Rowan Ranger (R3), Hubbard Red JA (A), CY Gen 5 × JA87 (CY), and M22 × JA87 (M)—were reared under organic conditions, fed ad libitum, and individually weighed weekly to calculate the daily weight gain (DWG). The behavioral observations consisted of the explorative attitude (EA), recorded at 21 days, and the behavioral patterns (BPs) recorded the week before the slaughter. The AS was established by a principal component analysis, and the AS of these genotypes was compared. Moreover, the effect of DWG and genotype on the AS was evaluated by univariable and multivariable regression models. Although the DWG and genotype were strictly dependent, genotype was the most important factor affecting the AS. In fact, its effect was significant both in univariable (*p* < 0.001) and multivariable models (*p* < 0.001). Conversely, the DWG was significant only in the univariable and lost significance when the effect of genotype was introduced in the model.

## 1. Introduction

In Europe, Commission Regulation No 848/2018 regulates organic systems for poultry and livestock production. This legislation provides some clarifications on several aspects of organic production, including animal feeding, presence of an outdoor run, importance of animal welfare, minimum slaughter age (81 days for chickens), and bans products obtained by chemical synthesis. The current regulation does not report details regarding the genotypes allowed in organic systems, since the use of slow-growing (SG) chicken strains is not compulsory. Moreover, there is not a definite criterion to classify whether a chicken is considered SG or fast growing (FG). The FG chickens are commonly represented by meat-type animals, used in the conventional rearing systems for meat production because they reach the commercial weight (2.8 kg) at about 42 days of age [1]. FG chickens have a higher live weight, breast yield, and feed efficiency than SG chickens [2]. In addition, the SG genotype showed 17% lower breast and drumstick yield than those of FG chickens when reared under the same conditions and at the same age [3]. Another aspect that distinguishes the SG genotypes from the FG ones is the body conformation, e.g., longer tibia and wings [4], which render SG genotypes generally more active and adapted to organic systems [5].

Concerning the SG group, it is necessary to specify that includes different kinds of animals such as the local strain chickens that are very important for maintaining biodiversity and genetic variability. However, they are not interesting from a commercial point of view due to their low productive performance (growth rate and breast yield) [6]. Furthermore, the SG group comprises also some commercial chicken strains, less performing compared with the FG, and for this reason, they are defined by breed companies as slower growing (SrG).

Castellini et al. [7] demonstrated a negative relationship between adaptability and daily weight gain (DWG) and affirmed that the SG genotypes appear more adapt to organic systems compared with their FG counterpart. However, within the same subcategories (e.g., SG), such relation is not confirmed. Accordingly, a study [6] showed that chicken genotypes with similar DWG exhibited different adaptability to the organic system, suggesting that genotype and growth rate are both key factors influencing the adaptability of animals.

According to the above-mentioned findings, the use of outdoor areas by the chickens depends on DWG, which affects the kinetic characteristics of birds, but it also relies on the innate behavior expressed by the chicken (i.e., exploratory attitude, use of outdoor space, etc.). Thus, considering that the organic system provides 4 m^2^/chicken of outdoor space, not all poultry genotypes are suitable to fully exploit outdoor areas, and therefore, the productive potential (grazing as a feed supplement, improvement of the immune system, and meat quality) of this system [8].

Therefore, the aim of this study was to develop an adaptability score (AS) for six SrG chickens organically reared, not based on the DWG only but resulting from a multifactorial approach that considers different parameters such as behavioral, plumage conditions, and body lesion indicators. The effect of DWG and genotype on the AS was evaluated with univariable and multivariable models.

## 2. Materials and Methods

### 2.1. Experimental Design

This research is part of an experimental trial defined by Cartoni Mancinelli et al., [6], in which animal housing is reported and described in detail. Briefly, the trial was conducted from March to May 2018 in the experimental facility of the University of Perugia according to EU Regulations 834/2007 and 889/2008 on animal welfare for experimental and other scientific purposes. A total of 600 male chickens from 6 different SrG genotypes were studied—namely, Ranger Classic (R1), Ranger Gold (R2), Rowan Ranger (R3), Hubbard Red JA (A), CY Gen 5 × JA87 (CY), and M22 × JA87 (M). Two breeders from their local hatcheries, Aviagen (Asti, Italy) and Hubbard (Quintin, France), provided the animals.

The test area consisted of six outdoor pens with the same dimensions (400 m^2^/pen), equipped with a shelter (10 m^2^), feeders, and drinkers each.

According to the organic rules, the animal density was 0.10 m^2^/bird indoor and 4 m^2^/bird outdoor. At the beginning of the trial (March), the six genotypes were separately located in the six shelters, and from 1 to 20 days old, chickens were reared indoors with a relative humidity between 65 and 70% and a temperature between 30 and 32 °C during the first week. Then, it decreased by 2 °C each week until it reached 24–26 °C. The chickens were individually weighed once a week (Mobile chicken scale 200 kg/5 g, Vignoli Forlì-Italy), and the daily weight gain (DWG) was calculated. At 18 days of age, 20 birds/genotypes, selected on the basis of the average weight of the group, were individually marked with different colors.

Outdoor space was available during the day for the chickens at 21 days old (April and May); the pasture was not treated with chemical substances or pesticides. During the trial in Perugia (Italy) in April and in May (months corresponding to the free access to outdoor space by the chickens) the maximum average temperature was 21 °C and minimum 10 °C. The birds were kept in the shelters during the night to protect them from predators. Chickens were fed ad libitum with the same diet, which was formulated integrating the nutritive recommendation of the breeding companies, the regulation of organic production (EU 889/2008), and the NCR [9] nutritional recommendations for broiler chickens, with particular consideration of crude protein (CP) and metabolizable energy (ME). The diet was divided into three periods:

- Starting period, 0–21 d, 24.01% CP, and 3245.20 Kcal/kg ME;
- Growing period, 22–60 d, 22.16% CP, and 3242.64 Kcal/kg ME;
- Finishing period, 61–81 d, 18.41% CP, and 3295.94 Kcal/kg ME.

Ingredients and chemical composition of diets are reported in Supplementary Materials (Table S1). At 81 days old, the birds were slaughtered 12 h after feed withdrawal in a commercial slaughterhouse according to the Regulation 1099/2009/EC for the protection of animals at the time of killing.

### 2.2. Behavioral Observations

Behavioral observations were performed using a computerized system (Noldus Technology, Wageningen, The Netherlands) consisting of two different software: Media Recorder to record the videos with the use of eight cameras and Observer XT to analyze the videos.

The behavioral observations for each genotype were divided into two different investigations: explorative attitude (EA) and behavioral patterns (BPs). The EA consisted of evaluating the time(s) required by animals to access the outdoor space in a fixed period (300 s [10]). Then, the EA score was calculated by normalizing the difference between the fixed maximum time and EA to a range of 0–1 (i.e., EA score = (300 − EA)/300)). A high EA score indicates a greater exploratory activity, while a score of 0 indicated that the animal had not accessed the outdoor space within the maximum time of 300 s. This parameter was recorded at 21 days when the shelter was opened for the first time, and it was repeated twice in the following days for a total of three acquisitions.

The BP was carried out by recording the behavior of the chickens at a 5 m distance from the shelter. This aspect was investigated at the end of the rearing cycle (1 week before the slaughtering day) to permit a better evaluation of the animal adaptability to the outdoor space. Cameras were positioned in advance on each pen in order to visualize all the space between the shelter and the distance of 5 m. From 74 to 81 days of chicken age, 3 videos of 20 min length each were recorded for each genotype by the remote activation of the cameras. The BP videos were processed by the Observer XT through the setup of a coding scheme with the different behaviors. The videos were then analyzed with the Observer XT by an expert observer through a pre-defined ethogram (Table 1) using the instantaneous scanning sampling method [11]. The behavior expressed from the 20 identified animals/genotypes, were analyzed individually. Thus, each animal represented an experimental unit, and the percentage of time dedicated to each specific behavior was calculated [6]. The behaviors recorded were divided into two main categories (Table 1): activity (walking, rest, roost) and feeding (feed, grass, drink).

### 2.3. Plumage Conditions and Body Lesions

In the 20 identified animals/genotypes at 81 days old, the evaluations of plumage conditions and body lesions were conducted by the same expert observer. The plumage score consisted of the average of the observations of five body regions (neck, chest, back, wings, and tails) performed according to the value scale presented in [12], between 0 (no feathering) and 4 (perfect plumage). The presence of body lesions (mainly sternal and footpad lesions) was evaluated on a scale between 0 (absence of lesions) and 1 to 2 (presence of light and deep ulcers, respectively), following the method reported by [13].

### 2.4. Statistical Analysis

Generalized linear models (GLMs) with identity link function and normal distribution were used to compare the mean DWG and BP between the genotypes. Sidak corrections were used for multiple comparisons. Kruskal–Wallis and Dunn’s multiple comparisons tests were instead used for the univariable analysis of the plumage and body lesion scores. Moreover, Spearman’s rho correlation was used to evaluate the associations between EA, BP, plumage, and body lesions score.

To define a score indicating the animal’s adaptability to the organic system, a principal component analysis (PCA) including behavioral variables, plumage conditions, and body lesion scores was performed. The variables were included in the PCA after inspection of the correlation matrix to identify very low or very high correlations [14,15], while Kaiser’s rule (eigenvalues > 1) was used to identify the number of factors [16]. Then, a principal component (PC) having eigenvalues > 1 was extracted describing multiple traits of adaptability [17]. Cronbach’s α assessed the reliability of this PC. Corresponding PC scores were calculated by the normalization (range 0,1) of the weighted sum scores. The scores represented each individual placement on the PC and created a new variable ranging from 0 to 1 called adaptability score (AS), which was used for subsequent analyses [18].

Associations between AS, genotype (R1, R2, R3, A, M, and CY), and DWG (continuous variable) were assessed using both uni- and multivariable approaches. First, AS was included as a dependent variable in two GLMs, separately analyzing the effect of DWG and genotype. Then, a multivariable regression model was used to evaluate the independent effects of DWG and genotype on AS. Multicollinearity was verified using variance inflation factors (VIFs [14]). Estimated parameters (Bwith standard error (SE) and *p-*values from the Wald test, were reported.

Statistical analyses were performed with SPSS Statistics version 25 (IBM, SPSS Inc., Chicago, IL, USA). The level for statistical significance was set at *p* < 0.05.

## 3. Results

### 3.1. Influence of Genotypes on Daily Weight Gain, Behavioral Parameters, and Plumage and Lesion Scores

**Figure 1:**
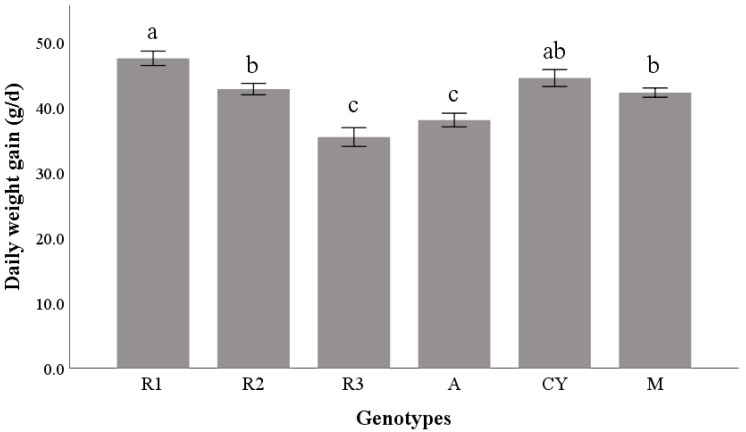
Daily weight gain (g/d) of six poultry genotypes on the entire rearing cycle. Abbreviations: R1 = Ranger Classic, R2 = Ranger Gold, R3 = Rowan Ranger, A = Hubbard Red JA, CY = CY Gen 5 × JA87, M = M22 × JA87. Bars not sharing any superscript letter are significantly different at *p* < 0.05 (Sidak correction).

Significant differences between strains were found in DWG (*p* < 0.001). R3 and A showed the lowest DWG, while the highest productive performance was achieved by R1 (Figure 1).

Genotype influenced the behavioral parameters (*p* < 0.001; Table 2). The M strains exhibited the lowest EA score and spent most of their time in “roost” behavior. Conversely, R3 and A had greater EA values, spending little time in rest and much more time walking and eating grass.

Finally, the non-parametric tests showed differences in both the plumage and body lesion scores (Table 2). The M strain showed the lowest plumage conditions, while CY presented the highest lesion score (*p* < 0.001).

The correlation among EA, behavior parameters (rest, walk, roost, feed, grass, and drink), and plumage and body lesions score (Table 3) evinced that the EA was positively correlated with walk and grass behaviors (0.813 and 0.809, respectively; *p* < 0.001) and negatively correlated with rest, roost, and feed behaviors (−0.291, −0.672 and −0.259, respectively; *p* < 0.001). Moreover, EA exhibited a positive correlation with the plumage score (0.658; *p* < 0.01), whereas was negatively correlated with the body lesions score (0.462; *p* < 0.01).

### 3.2. Principal Component Analysis

The EA score, activity and eating behaviors, the median value of plumage scores, and total score of body lesions were initially included in the PCA. After inspection of the correlation matrix, drink and feed activities were eliminated because they were poorly correlated with the other variables (all correlation coefficients r < 0.04). The PCA extracted a PC with eigenvalues greater than 1, which explained more than 50% of the variance and showed good reliability (α > 0.7). In this PC, high positive loadings were observed for EA score, walking, grass intake, and high total plumage score. Conversely, negative loadings were found for static behaviors (roost and rest), and the presence of body lesions (Table 4). PC scores were calculated for each subject creating a new variable called adaptability score (AS).

### 3.3. Effect of Genotype and Daily Weight Gain on the Adaptability Score

**Figure 2:**
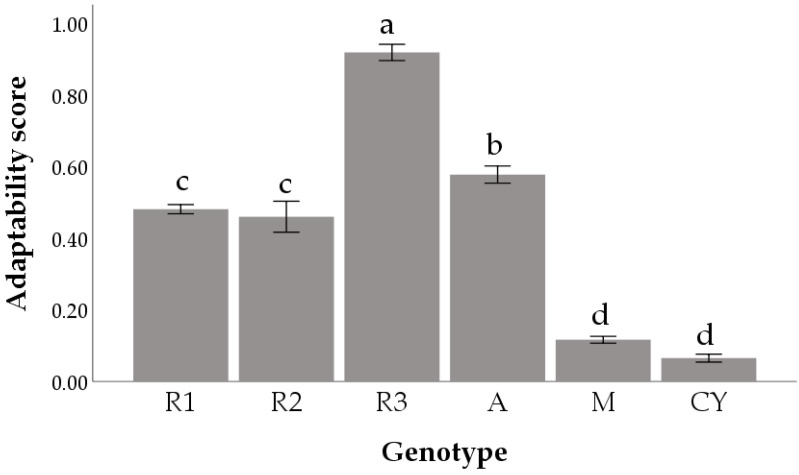
Adaptability score (mean ± SE; raw data) of six poultry genotypes. Abbreviations: R1 = Ranger Classic, R2 = Ranger Gold, R3 = Rowan Ranger, A = Hubbard Red JA, CY = CY Gen 5 × JA87, M = M22 × JA87. Bars not sharing any superscript are significantly different at *p* < 0.05 (Pairwise comparisons with Sidak correction).

Genotype strongly influenced AS, and statistical significance was observed both by uni- and multivariable analyses (i.e., adjusting for DWG; *p* < 0.001; Table 5). This finding indicates that genotypes influence AS even if the DWG is held constant. Pairwise comparisons showed that the R3 strain had the greatest scores, while CY showed the lowest scores (Figure 2).

The effect of DWG was significant in the univariable analysis (Table 5), and the B parameter suggests that when DWG increases, AS decreases (B= −0.029; *p* < 0.001; Table 5). However, DWG lost significance in the model in which the genetic strain was included (i.e., after adjusting for genotype: *p* = 0.156). This finding suggests that the effect of DWG on AS is not independent of the genotype.

## 4. Discussion

The choice of suitable genotypes for the organic system is still an open question, and currently, the DWG is considered the main aspect to be considered. This approach paradoxically allows the use of FG genotypes submitted to specific rearing strategies (feed restriction, use of female chickens) to reduce the DWG [8]. It is well known that FG chickens reach the commercial weight in 35–42 days, which corresponds to roughly half the time requested by organic system regulations in Europe (81 days). In fact, the FG strains have shown lower survival rates in organic systems, at 65 days, compared with SG chickens, and 90% of this mortality was due to sudden death syndrome [3]. Moreover, FGs reared in the organic system exhibited many welfare problems such as a higher occurrence of footpad dermatitis and breast blisters, as well as an impaired immune response, compared with SG genotypes [19].

For these reasons, it is important to define the adaptability of poultry for organic systems by considering the DWG, but also including welfare, EA, and BP parameters.

To our knowledge, this is one of the few studies that adopted a multifactorial method to assess the adaptability of poultry genotypes to organic systems. The six genotypes considered in this study, although defined SrR by the breed companies, showed strong differences concerning DWG, EA, BP, plumage conditions, and body lesions, when reared in an organic system. In particular, CY and R1 exhibited the highest DWG, followed by M and R2, whereas A and R3 had the lowest growth rates.

In particular, the behavioral observations, and the plumage and body lesion scores revealed a strict connection with EA. In fact, our data indicate that the less explorative genotypes (CY and M) also had a higher percentage of static behaviors and worst plumage conditions and body lesions. On the contrary, genotypes having greater EA scores (R3 and A) were more active animals for the organic system such as walking and grass intake activities.

This is consistent with our previous studies [6,7] showing that the longer the EA time is, the lesser the ability of the genotype will be to use the outdoor space. Indeed, the SG genotype has been shown to be the most active genotype, both in conventional and organic systems; however, basic active behaviors (walking, ground pecking, wing flapping, etc.) increased when the SG birds were reared in an organic system, indicating that they are more suitable to be reared in the presence of outdoor runs [20].

The behavioral observations (Table 2) revealed a strict connection between EA and BPs; CY and M exhibited a lower EA and spent most of their budget time in static activities such as “roost” (67% and 62%, respectively) and “rest” (33% and 25%, respectively). Conversely, A and R3 birds had lower EA times (48 s and 43 s, respectively) and appeared to be more active animals. The R3 birds spent most of their time eating grass (63.7%) and A birds showed variability among the BP activities (18.3% “rest”, 26.5% “walk”, 29.5% “roost”, 25.7% “grass”).

As mentioned above, in this study, different variables such as EA, BP, plumage conditions, and body lesion indicators were used to develop the AS. A high AS indicates good adaptability, which implicitly means that the animal spent a considerable time eating grass, walking, and also showing a high plumage condition and a good EA score. Conversely, animals with a low AS were characterized by a worst EA score, with more time spent resting and roosting, as well as by the presence of body lesions. All these factors suggested poor adaptability. Additionally, from a metabolic point of view [21], SG and FG showed different adaptability to walking activity: the SG strain exhibited a progressive stress adaptation to exercise (daily moderate locomotory activity for 1 h at 4 km/h), with an improvement in the blood oxidative status due to body training, whereas the FG birds were not able to counteract the free radical production induced by walking activity [22].

However, our data showed that DWG also affected the active animal behaviors: CY, which was the genotype with the highest DWG, was the most static group, while the animals belonging to the R3 group, which showed the lowest growth rate, appeared to be the most active birds. Notoriously, the active nature of chickens is negatively related to the DWG and slaughter age. Previous studies reported that the FG genotype spent 79% of their time lying down between 39 and 49 days of age, and their walking activity (3.3% of the time) decreased with increasing age [23]. The plumage conditions and body lesions confirmed that the more static genotypes (M and CY) also exhibited the lowest scores. Accordingly, it has been reported that these body lesions are associated with the contact of the body, mainly the plumage of the breast and the feet, with the humid and soiled bedding [24].

In this study, the greatest AS was shown by the R3, followed by A, R2, and R1, whereas the CY and M strains (the chickens with the highest DWG) seemed to not be suitable for the organic system. DWG is confirmed as an important factor affecting most of the analyzed parameters (BP, welfare); however, it cannot be the driving force for assuring suitable adaptability to organic systems.

Indeed, in our study, the DWG was significant only in the univariable but, when the effect of genotype was included in the model (multivariable), it lost significance. This could explain why animals with the same DWG showed different behaviors and levels of adaptability to organic systems [6].

**Figure 3:**
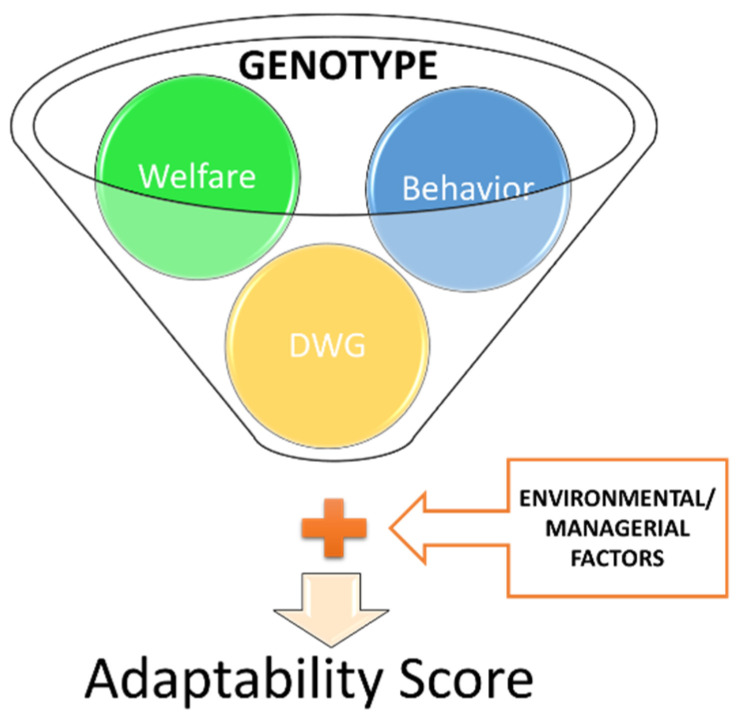
Graphical representation of the influence of the main parameters investigated on the AS and their interaction.

Our study suggests that the genotype is a combining of specific traits such as: welfare, behavior as well as productive performance (DWG), which interacting with environmental and managerial factors affect the chickens AS (Figure 3). The main factor that influenced the adaptability score was represented by the genotype, which, in turn, was characterized by behavior, welfare, and daily weight gain (DWG). All of these parameters are influenced by the environment and the adopted management.

## 5. Conclusions

The proposed adaptability score, based on the evaluation of behavioral and welfare traits, estimates the chicken adaptability by the use of a multifactorial approach, and it could represent a useful tool to identify the suitability of chicken strains to the organic system. This study reveals a strict relation between DWG and genotype, but the latter is the most important factor affecting the AS of poultry strains.

Thus, it is possible to define the genotype as an independent predictor of adaptability that should be considered when selecting poultry strains suitable to the organic system, whereas DWG represents a “related predictor” with a moderate accuracy for adaptability that could be used in order to exclude the use of fast-growing genotypes in the organic system. This paper could be considered a first step to identifying the main factors involved in poultry adaptability. However, further studies are needed to better highlight the inherent relationships between welfare, behavioral patterns, and DWG, and to clarify the role of each parameter on AS.

## Figures and Tables

**Table 1:** Main behavioral patterns expressed by the chickens at 5 m from the shelter.

Behavior Category	Behaviors	Description
**Activity**	Walking	Bird that moves more than three steps.
	Rest	Bird that presents the body in line with the ground with an erect head and open eyes.
	Roost	Bird in lying position with the ventral body region in contact with the floor.
**Eat**	Feed	Bird that pecks inside the feeder.
	Grass	Bird that presents its head down and beak in contact with the grass.
	Drink	Bird that pecks the drinker.

**Table 2:** Explorative attitude (EA, score), behavioral patterns (BPs, in percentage), and plumage and lesion scores (scale 0–4) expressed by the six genotypes studied.

Parameters		Genotypes						SE est.	*p* Value *
		R1	R2	R3	A	CY	M		
EA score		0.82 ^b^	0.83 ^c^	0.84 ^d^	0.86 ^e^	0.00 ^a^	0.00 ^a^	0.10	<0.001
BP ^†^	Rest	12.1 ^cd^	23.2 ^bc^	9.0 ^d^	18.3 ^bcd^	33.0 ^a^	25.2 ^ab^	3.1	<0.001
	Walk	15.8 ^b^	11.4 ^b^	26.3 ^a^	26.5 ^a^	0.0 ^c^	0.0 ^c^	2.6	<0.001
	Roost	28.5 ^b^	26.8 ^b^	1.0 ^c^	29.5 ^b^	67.0 ^a^	62.8 ^a^	2.9	<0.001
	Feed	0.0 ^b^	13.8 ^a^	0.0 ^b^	0.0 ^b^	0.0 ^b^	12.0 ^a^	1.1	<0.001
	Grass	10.6 ^c^	24.8 ^b^	63.7 ^a^	25.7 ^b^	0.0 ^d^	0.0 ^d^	3.4	<0.001
	Drink	33.0 ^a^	0.0 ^b^	0.0 ^b^	0.0 ^b^	0.0 ^b^	0.0 ^b^	0.7	<0.001
	Plumage ^‡^	4.0 ^a^ (3.0–4.0)	4.0 ^a^ (3.0–4.0)	3.5 ^a^ (3.0–4.0)	3.5 ^a^ (3.0–4.0)	3.0 ^ab^ (2.0–3.0)	2.0 ^b^ (2.0–3.0)	-	<0.001
	Body Lesions ^‡^	1.0 ^b^ (0.0–1.0)	0.0 ^a^ (0.0–1.0)	0.0 ^a^ (0.0–1.0)	0.0 ^a^ (0.0–0.0)	2.0 ^c^ (2.0–3.0)	0.0 ^a^ (0.0–0.0)	-	<0.001

Abbreviations: R1 = Ranger Classic, R2 = Ranger Gold, R3 = Rowan Ranger, A = Hubbard Red JA, CY = CY Gen 5 × JA87, M = M22 × JA87. SE est = standard error of the estimate. * univariable analysis; † in percentages, mean ± SE; ‡ scale 0–4, median (IQR); values in the same row not sharing the same superscript are significantly different at *p* < 0.05 (Sidak correction or Dunn’s multiple comparisons test).

**Table 3:** Correlation (Spearman’s rho) among explorative aptitude score (EA score), behavior (%, rest, walk, roost, feed, grass, and drink), and welfare (plumage and body lesion score) parameters.

	Rest	Walk	Roost	Feed	Grass	Drink	Plumage Score	Body Lesions Score
EA Score	−0.291 *	0.813 **	−0.672 **	−0.259 *	0.809 **	−0.084	0.658 **	−0.426 **
Rest		−0.483 **	0.376 **	0.205	−0.492 **	−0.335 **	−0.192	0.168
Walk			−0.735 **	−0.427 **	0.741 **	0.130	0.540 **	−0.288 *
Roost				0.296 *	−0.847 **	−0.118	−0.528 **	0.175
Feed					−0.263 *	−0.306 *	−0.278 *	−0.243
Grass						−0.094	0.577 **	−0.281 *
Drink							0.232	0.107
Score								−0.274 *

* Correlation is significant at the 0.05 level (two-tailed); ** Correlation is significant at the 0.01 level (two-tailed).

**Table 4:** Loadings of the variables included in the principal component analysis.

Item	Loading
EA score	0.928
Walking	0.780
Eating grass	0.775
Plumage score	0.732
Body lesions	−0.546
Rest	−0.546
Roost	−0.903
% Variance explained	58.3
Eigenvalue	4.082
Cronbach’s α	0.738

EA score, explorative aptitude score.

**Table 5:** Factors affecting the adaptability score: results of uni- and multivariable analyses.

Factor	Univariable ^#^			Multivariable *		
	B	SE	*p* Value	B	SE	*p* Value
Daily weight gain	−0.029	0.0064	**<0.001**	−0.004	0.0008	0.156
Genotypes			**<0.001**			**<0.001**
R1 vs. CY	0.416	0.0301	**<0.001**	0.427	0.0306	**<0.001**
R2 vs. CY	0.395	0.0309	**<0.001**	0.389	0.0307	**<0.001**
R3 vs. CY	0.855	0.0301	**<0.001**	0.821	0.0377	**<0.001**
A vs. CY	0.513	0.0301	**<0.001**	0.490	0.0339	**<0.001**
M vs. CY	0.051	0.0301	0.088	0.043	0.0302	0.152

Abbreviations: R1 = Ranger Classic, R2 = Ranger Gold, R3 = Rowan Ranger, A = Hubbard Red JA, CY = CY Gen 5 × JA87, M = M22 × JA87, B = parameter estimated, SE= standard error; dependent variable: adaptability index; ^#^ model for daily weight gain: (intercept), daily weight gain; model for genotypes: (intercept), genotypes; * model: (intercept), daily weight gain, genotypes. Values in bold are statistically significant at the *p* < 0.001.

## Data Availability

None of the data were deposited in an official repository.

## Supplementary Materials

The following are available online at https://www.mdpi.com/article/10.3390/ani11102992/s1, Table S1. Ingredients (%) and chemical composition of the three experimental diets.
