# Supplementary material for: The Assessment of a Multifactorial Score for the Adaptability Evaluation of Six Poultry Genotypes to the Organic System
Source: Animals (Basel). 2021 Oct 18;11(10):2992. doi: 10.3390/ani11102992 (PMC8532737; doi:10.3390/ani11102992)
Supplement: Supplementary file 1 [file animals-11-02992-s001.zip › animals-1399718-supplementary.pdf]

Supplementary material

Table S1. Ingredients (%) and chemical composition of the three experimental diets

**Ingredients (%)**		**Starter**	**Grower**	**Finisher**
Corn		53.92	55.95	53.11
Soybean flour extract 48%		30.23	24.67	15.69
Extruded corn flour		5.08	8.90	11.45
Common wheat		5.00	5.00	15.00
Dicalcium phosphate		1.71	1.58	1.21
Calcium carbonate		1.23	1.16	1.29
Corn gluten 70		1.00	1.00	
Soybean oil		0.62	0.54	1.15
Vitamin supplement		0.40	0.40	0.40
salt		0.20	0.18	0.23
Mineral supplement		0.16	0.16	0.11
Sodium bicarbonate		0.15	0.15	0.15
Chemical composition (% s.s.)				
Humidity	%	12.20	12.11	12.00
Crude protein	%	24.01	22.16	18.41
Lipids	%	3.99	3.98	4.55
Crude fiber	%	3.48	3.58	3.60
Ashes	%	6.92	6.43	5.78
Metabolizable energy	kcal/kg	3245.20	3242.64	3295.94
VIT A	U.I.	11385.93	11377.59	11364.80
VIT E	Mg	36.43	36.4	36.37
